# Glycemic control and neonatal outcomes in women with gestational diabetes mellitus treated using glyburide, metformin, or insulin: a pairwise and network meta-analysis

**DOI:** 10.1186/s12902-021-00865-9

**Published:** 2021-10-12

**Authors:** Dan-Qing Yu, Guan-Xin Xu, Xin-Yuan Teng, Jing-Wei Xu, Liang-Fang Tang, Chun Feng, Jin-Peng Rao, Min Jin, Li-Quan Wang

**Affiliations:** The Second Affiliated Hospital, School of Medicine, Zhejiang University, 88 Jiefang Rd, Zhejiang, 310009 Hangzhou China

**Keywords:** Network meta-analysis, Gestational diabetes mellitus, Metformin, Glyburide, Insulin

## Abstract

**Aims:**

We aimed to assess the comparative efficiency and safety of the use of glyburide, metformin, and insulin in gestational diabetes mellitus (GDM).

**Methods:**

We searched for randomized controlled trials that compared glyburide, metformin, and insulin in GDM. Data regarding glycemic control and neonatal safety were collected and analyzed in pairwise and network meta-analyses.

**Results:**

A total of 4533 individuals from 23 trials were included. Compared with glyburide, metformin reduced 2-h postprandial blood glucose (2HPG) to a greater extent (standard mean difference (SMD) 0.18; 95% credible interval (CI) 0.01, 0.34). There were significantly lower prevalence of neonatal hypoglycemia (risk difference (RD) − 0.07; 95%CI − 0.11, − 0.02) and preeclampsia (RD − 0.03; 95%CI − 0.06, 0) in the metformin group than in the insulin group. The metformin group had significantly lower birth weight (SMD − 0.17; 95%CI − 0.25, − 0.08) and maternal weight gain (SMD − 0.61; 95%CI − 0.86,− 0.35) compared with the insulin group. Network meta-analysis suggested that metformin had the highest probability of successfully controlling glycemia and preventing neonatal complications.

**Conclusions:**

The present meta-analysis suggests that metformin may be as effective as insulin for glycemic control and is the most promising drug for the prevention of neonatal and maternal complications.

**Supplementary Information:**

The online version contains supplementary material available at 10.1186/s12902-021-00865-9.

## Introduction

Gestational diabetes mellitus (GDM) is defined in the World Health Organization guidelines as glucose intolerance or hyperglycemia that occurs or is first recognized during pregnancy. Owing to changes in lifestyle and increasing maternal age, the prevalence of GDM has increased markedly during the past two decades, with prevalences of between 1 and 26% being recorded in different studies [1–6]. Especially, a high prevalence of GDM among the Asia population has been reported, ranging from 0.7 to 51.0% [7–9]. A recent meta-analysis reported the overall prevalence of GDM in Asia is 11.5% [10].

Poorly managed GDM can lead to maternal and neonatal adverse outcomes in both the short and long term. The maternal complications include preeclampsia (PE), cesarean section, and polyhydramnios in the short term, and the progression of diabetes mellitus after pregnancy in the long term [1]. The fetal and neonatal complications include congenital malformation, neonatal death, stillbirth, macrosomia, obstetric trauma, shoulder dystocia, and neonatal hypoglycemia [11, 12]. A recent study reported the risks of preterm birth (OR 1.3 [95% CI 1.3, 1.4]), macrosomia (OR 1.8 [95% CI 1.7, 1.8]), respiratory distress (OR 1.1 [95% CI 1.0, 1.3]), birth trauma (OR 1.3 [95% CI 1.1, 1.5]) and cardiac malformations (OR 1.3 [95% CI 1.1, 1.4]) [13].

Appropriate glycemic control is the principal means of preventing hyperinsulinemia and macrosomia [14]. As the conventional treatment for GDM, insulin is effective and safe. However, it is inconvenient and expensive to use. During the last 20 years, oral hypoglycemic agents (OHAs) have been introduced for GDM, of which the most frequently used drugs are glyburide and metformin. Glyburide is a second-generation sulfonylurea that binds to receptors on beta-cells and increases the secretion of insulin [15]. Metformin is a biguanide that increases insulin-stimulated glucose uptake in muscle cells, and suppresses gluconeogenesis and fatty acid oxidation in hepatocytes [16]. Because they are cheap and convenient, OHAs have been used by increasing numbers of patients and health care providers. In the USA, glyburide has been widely used for the treatment of GDM, whereas in Europe it has not been routinely used to treat GDM [17]. Metformin is labeled as a category B drug, meaning that there is no strong evidence that it causes primary fetal malformation [18]. Therefore, it has been commonly used for the treatment of polycystic ovary syndrome [19, 20]. Although there is an increasing amount of evidence that supports the use of glyburide or metformin for GDM, the American Diabetes Association (ADA) and American College of Obstetricians and Gynecologists (ACOG) still recommend insulin as the primary medical treatment if the glycaemic treatment goals are not achieved with life-style intervention, due to the lack of evidence regarding the long-term safety of the alternatives [21, 22].

Both glyburide and metformin can pass across the placenta [18, 23, 24], and therefore there is potential for adverse effects on the fetus. Recent studies and meta-analyses have shown higher prevalences of neonatal complications, mainly macrosomia and neonatal hypoglycemia, in users of glyburide [25–32], and higher prevalences of preterm birth and preeclampsia in users of metformin [33–36], compared with users of insulin.

In 2019, two pairwise meta-analyses were conducted of glycemic control and neonatal outcomes. Both of these showed significant heterogeneity in glycemic control [37, 38]. However, because these studies used blood glucose concentration in the third trimester as the index of glycemic control, the heterogeneity may at least, in part, have been due to the differing baseline glucose concentrations in different populations and among individuals with different lifestyles. In the present study, we calculated the changes in FBG, 2HPG, and HbA1c that occurred from baseline during treatment. This approach was intended to limit the heterogeneity, such that the indices would more accurately reflect the effects of treatment.

The aim of the present study was to identify all the RCTs that have compared the use of glyburide, metformin, and insulin to treat GDM and conduct a network meta-analysis to compare the effects of each on glycemic control and neonatal outcomes, as well as their safety. In this way, we aimed to provide evidence for the rational treatment of GDM in clinical practice.

## Research design and methods

We conducted this study according to the *Cochrane Handbook for the Systematic Review of Interventions* and the *Guidelines of the Preferred Reporting Items for Systematic Review and Meta-Analysis Protocols (PRISMA, version 6)*. This meta-analysis was registered with PROSPERO (number CRD42020178575).

### Ethics

The ethics committee of the Second Affiliated Hospital Zhejiang University School of Medicine ruled that no formal ethics approval was required in this meta-analysis, as the data used are all publicly available.

### Inclusion and exclusion criteria

We considered RCTs of GDM that compared any of the following treatments: glyburide, metformin, and insulin. Trials of dietary control and exercise may have included participants with differing baseline blood glucose concentrations, and were therefore not considered. Literature searches were conducted in PubMed, Web of Science, EBSCO, and Scopus. Two assessors screened the publications independently on April 7, 2020. The search terms used were ‘gestational diabetes’ or ‘GDM’; ‘insulin’, or ‘oral hypoglycemic agents’, or ‘oral antidiabetic drugs’, or ‘glibenclamide’, or ‘glyburide’, or ‘metformin’; and ‘randomized controlled trial’. The searches were limited to English language publications. We also checked the reference lists of the included studies for further relevant trials.

Trials with patients developed diabetes before gestation were excluded. Trials that only included patients with mild GDM were also excluded; this is because we were concerned about the effects of pharmacotherapy in patients with poor glycemic control after medical nutritional therapy. Furthermore, studies were excluded that contained only per-protocol (PP) analyses, as different administration routes of insulin and OHAs results in different compliance. Moreover, patients failed in OHAs groups are usually more severe patients, who are excluded in PP analyses. Studies with the baseline characteristics not well-matched were excluded, except for glycemic control parameters.

### Assessment of the risk of bias in the included studies

Two authors independently assessed the risk of bias using the Cochrane Handbook for Systematic Reviews of Interventions. Any disagreement between the authors was resolved by discussion.

### Outcomes

Estimates of the mean changes and standard deviations from baseline of FBG, 2HPG, and HbA1c were reported as effects of the treatments on glycemic control. They were calculated using the means and standard deviations of the baseline and post-treatment values, using the recommended approach [39, 40]. Briefly, SD (change) was estimated as the square of (SD (baseline)^2^ + SD (final level)^2^-(2*R1*SD(baseline)*SD(final level))).

The neonatal outcomes were anomalies, neonatal hyperbilirubinemia, neonatal hypoglycemia, neonatal intensive care unit (NICU) admission, respiratory distress syndrome (RDS), obstetric trauma, preterm birth, perinatal death, birth weight, macrosomia, large-for-gestational age (LGA) fetus, and small-for-gestational age (SGA) fetus. The maternal outcomes were preeclampsia and maternal weight gain from the time of enrollment.

### Data synthesis and statistical analysis

In trials that reported findings in participants who were treated with metformin alone or metformin and supplementary insulin, the mean and SD of the metformin group was calculated from the data from the divided subgroups, as previously reported [41]. Glyburide- and glibenclamide-treated participants were considered together in the present analysis.

Pairwise meta-analysis was conducted using Review Manager (version 5.3). We conducted pairwise meta-analysis with a random-effect model if significant heterogeneity was identified; otherwise, a fixed-effect model was used. The Studies were determined to be heterogeneous if I^2^ > 50% and *P* < 0.1. The standard mean difference (SMD) was calculated using the mean and SD for continuous variables and the risk difference (RD) was calculated for dichotomous variables. Both are quoted with 95% credible intervals (CIs).

Glycemic control and neonatal and maternal safety outcomes were synthesized using network meta-analysis (NMA) in a Bayesian multilevel framework. NMA was performed using Stata (version 15). We present the results as a ranking probability, using their surface under the cumulative ranking curve (SUCRA), for different efficiency and safety outcomes. For each network analysis, we assessed the consistency of direct and indirect comparisons using node-splitting analysis, with *p* < 0.05 indicating significant inconsistency. The results indicated that the direct and indirect comparisons were consistent for all the NMAs, and the results of the NMAs are shown using consistency models.

## Results

### Characterization of the included trials

A total of 1708 records were identified through the database searches, of which 1196 records remained after the removal of duplicates. Thirty-four studies met the inclusion criteria, of which four included duplication with regard to follow-up data [42–45]. Therefore, the later publications were not included in the final analysis. The study and patient characteristics of the 30 remaining studies are summarized in Table 1. The screening protocols and diagnostic criteria for GDM, and the details of the funding and conflict of interest are summarized in Supplementary Table 1 [33, 34, 36, 45–71]. Insulin was most frequently prescribed as a combination of rapid-acting and intermediate insulin (Supplementary Table 1). The most frequently used targets for glucose control were FBG < 90–100 mg/dl and 2HPG < 120–126 mg/dl (Supplementary Table 1). Of these 31 studies, four reported data according to the per-protocol (PP) principle [55, 61, 65, 70] and one did not report the principle of the data analysis [47]. Three studies reported results according to both the intention-to-treat (ITT) and PP principles [49, 50, 63] because no patient drop out from the group after randomization, and one study reported both ITT and PP data [69]. Therefore, the four studies that were conducted according to an unknown principle or only used the PP principle were not included in quantitative analysis. As shown in Table 1, one study showed a significant difference in maternal age between the groups at baseline [63] and another showed a significant difference in gestational age at enrollment [51], which were not included in the meta-analysis because these two parameters have direct effects on the efficacy of treatment. A PRISMA flow chart that summarizes the search results and the trials included in the analysis is provided in Supplementary Fig. 1. The risks of bias in the 24 studies included in the meta-analysis are summarized in Supplementary Figs. 2 and 3.

**Table 1:** Characteristics of the included trials

Study (author year)	Country	Principle of data analysis	Drug		Sizes		BMI		Age		Gestational age at enrollment		Included in meta analysis
			Group 1	Group 2	Group 1	Group 2	Group 1	Group 2	Group 1	Group 2	Group 1	Group 2	
Rowan 2003	NA	NA	Metformin	Insulin	16	14	39.5 ± 6.94	37.9 ± 6.87	33.7 ± 4.44	34.1 ± 3.7	29.8 ± 4.49	30.4 ± 4.67	No
Moore 2007	USA	ITT/PP	Metformin	Insulin	32	31	39.7 ± 9	35.3 ± 6.7	27.1 ± 4.7	27.7 ± 6.7	27.8 ± 6.5	28.9 ± 5	Yes
Rowan 2008	New Zealand, Australia	ITT	Metformin	Insulin	363	370	35.1 ± 8.3	34.6 ± 7.2	33.5 ± 5.4	33 ± 5.1	30.2 ± 3.3	30.1 ± 3.2	Yes
Ijas 2010	Finland	ITT	Metformin	Insulin	47	50	31.5 ± 6.5	30.8 ± 5.4	32.3 ± 5.6	31.7 ± 6.1	30 ± 4.9	30 ± 4	Yes
Niromanesh 2012	Iran	ITT	Metformin	Insulin	80	80	28.1 ± 4	27.1 ± 2.1	30.7 ± 5.5	31.8 ± 5.1	28.7 ± 3.7	28.6 ± 3.6	Yes
Mesdaghinia 2012	Iran	PP	Metformin	Insulin	100	100	27.6 ± NA	28.46 ± NA	29.6 ± 5.3	30.2 ± 5.9	27.9 ± 3.22	28.9 ± 3.8	No
Hassan 2012	Pakistan	ITT	Metformin	Insulin	75	75	29.17 ± 1.94	28.74 ± 2.69	30.29 ± 3.06	30.88 ± 3.6	29.53 ± 1.33	29.2 ± 1.48	Yes
Spaulonei 2013	Brazil	ITT	Metformin	Insulin	46	46	31.96 ± 4.75	31.39 ± 5.71	31.93 ± 6.02	32.76 ± 4.66	32.18 ± 3.7	32.05 ± 3.5	Yes
Tertti 2013	Finland	ITT	Metformin	Insulin	110	107	29.4 ± 5.9	28.9 ± 4.7	31.9 ± 5	32.1 ± 5.4	30.3 ± 2	30.4 ± 1.8	Yes
Ruholamin 2014	Iran	PP	Metformin	Insulin	50	50	26.4 ± 2.8	25.1 ± 3.4	24.6 ± 6.3	23.4 ± 2.5	27.6 ± 3.3	26.7 ± 3.5	No
Ashoush 2016	Egypt	ITT	Metformin	Insulin	47	48	31.1 ± 1.3	31.4 ± 1.5	31.6 ± 2.8	32.1 ± 3.2	29.8 ± 1.4	29.7 ± 1.9	Yes
Saleh 2016	Egypt	ITT	Metformin	Insulin	67	70	30.52 ± 3.17	31.58 ± 30.12	31 ± 3.42	29.8 ± 2.18	NA	NA	Yes
Arshad 2017	Pakistan	ITT	Metformin	Insulin	25	25	NA	NA	29.76 ± 3.41	31.6 ± 4.27	NA	NA	Yes
Gamal 2018	Egypt	ITT	Metformin	Insulin	58	58	29.6 ± 1.3	29.4 ± 1.4	30.4 ± 2.8	30.6 ± 2.5	28.9 ± 1.1	29 ± 1.1	Yes
Ghomian 2019	Iran	PP	Metformin	Insulin	143	143	NA	NA	NA	NA	NA	NA	No
Wasim 2019	Pakistan	ITT	Metformin	Insulin	137	141	NA	NA	29.5 ± 4.8	29.7 ± 4.8	28.9 ± 2.9	28.6 ± 3.1	Yes
Langer 2000	USA	ITT	Glyburide	Insulin	201	203	NA	NA	29 ± 7	30 ± 6	24 ± 7	25 ± 7	Yes
Bertini 2005	Brazil	ITT	Glyburide	Insulin	24	27	27.5 ± 5.8	27 ± 7.2	31.2 ± 4.5	28.7 ± 6	NA	NA	Yes
Anjalakshi 2007	India	ITT/PP	Glyburide	Insulin	10	13	22.82 ± 3.5	25.32 ± 5.14	24.9 ± 3.76	27.46 ± 5.83	22.5 ± 4.72	22.62 ± 5.62	Yes
Ogunyemi 2007	USA	ITT	Glyburide	Insulin	48	49	32 ± 7.6	30.8 ± 6.9	NA	NA	28.1 ± 7.6*	24.6 ± 8*	No
Lain 2009	USA	ITT	Glyburide	Insulin	41	41	33.4 ± 12.9	30.9 ± 5.7	32.2 ± 5	31.2 ± 5.9	30.8 ± 2.5	30.6 ± 2.2	Yes
Mukhopadhyay 2012	India	ITT	Glyburide	Insulin	30	30	23.7 ± 2.7	23 ± 2.9	26.3 ± 4.6	26 ± 4.3	28.3 ± 2.2	27.4 ± 2.7	Yes
Tempe 2013	India	ITT	Glyburide	Insulin	32	32	NA	NA	NA	NA	NA	NA	Yes
Mirzamoradi 2015	Iran	ITT/PP	Glyburide	Insulin	37	59	NA	NA	29.5 ± 4.06**	31.18 ± 5.01**	29.89 ± 4.12	30.27 ± 3.97	No
Bhrashi 2016	Iran	PP	Glyburide	Insulin	120	129	21.94 ± 2.8	22.59 ± 3.094	30.69 ± 7.194	29.98 ± 7.033	24.89 ± 3.9	24.48 ± 4.51	No
Senat 2018	France	PP/ITT	Glyburide	Insulin	448(ITT)	442	30.7 ± 5.1	31.1 ± 5.4	32.5 ± 5.1	32.6 ± 5.3	NA	NA	Yes
Moore 2009	USA	ITT	Glyburide	Metformin	74	75	32.7 ± 7	32.8 ± 5.8	29.6 ± 7.8	31 ± 7.1	29.1 ± 5	27.3 ± 6.8	Yes
Silva 2012	Brazil	ITT	Glyburide	Metformin	96	104	28.61 ± 5.88	28.68 ± 5.37	31.29 ± 5.36	32.63 ± 5.61	25.44 ± 7.13	26.96 ± 6.44	Yes
George 2015	India	ITT	Glyburide	Metformin	53	51	28.8 ± 4	28.7 ± 4.4	33.6 ± 4.6	33.4 ± 4.4	29.7 ± 3.7	29.3 ± 3.3	Yes
Nachum 2017	Israel	ITT	Glyburide	Metformin	53	51	28.6 ± 4.7	28.6 ± 5.5	32.8 ± 5	33.6 ± 5.3	29.4 ± 4	29.6 ± 4.1	Yes

ITT intention-to treat, PP per-protocol, BMI body mass index, NA not available

** *p* value < 0.01, * *p* value< 0.05, marked in bold

### Glycemic control

Of the 23 studies that were quantitatively analyzed, 20 reported both baseline and final glucose levels. The changes from baseline were calculated using the reported baseline and post-treatment blood glucose concentrations for ITT studies (Table 2). One showed significantly lower baseline FBG in the metformin group than in the insulin group (104 ± 13.12 mg/dl vs. 117.9 ± 29.06 mg/dl) [67]. Furthermore, Mukhopadhyay et al. reported significantly lower baseline 2HPG (184.1 ± 20.46 mg/dl vs. 194.3 ± 18.47 mg/dl) levels in the glyburide group compared with the insulin group [57]. However, both studies were included in the meta-analysis because we calculated the changes from baseline for FBG, 2HPG, and HbA1c. To minimize the possible influence of imbalanced baseline, we also did meta-analysis after removal of these two studies in supplementary Fig. 5, 7 and 9.

**Table 2:** Baseline and effect of treatment on FBG, 2HPG and HbA1c

Study (author year)	Drug	FBG			2HPG			HbA1c		
		Baseline (SD)	After treatment (SD)	Change from baseline (SD)	Baseline (SD)	After treatment (SD)	Change from baseline (SD)	Baseline (SD)	After treatment (SD)	Change from baseline (SD)
Rowan 2008	Metformin	102.6 (21.6)	93.6 (10.8)	−9 (18.7)	174.6 (37.8)	111.6 (10.8)	−63 (33.72)	5.7 (0.6)	5.6 (0.5)	−0.1 (0.557)
	Insulin	102.6 (19.8)	91.8 (12.6)	−10.8 (17.36)	169.2 (37.8)	115.2 (16.2)	−54 (32.85)	5.8 (0.7)	5.7 (0.6)	−0.1 (0.656)
Niromanesh 2012	Metformin	104.7 (8.6)	88.3 (7.7)	−16.4 (8.19)	164.6 (28.6)	111.3 (9.1)	−53.3 (25.31)	5.7 (0.6)	4.3 (0.5)	−1.4 (0.56)
	Insulin	107.1 (9.2)	88.7 (6.3)	−18.4 (8.15)	172.5 (30)	111.1 (9)	−61.4 (26.66)	5.6 (0.7)	4.3 (0.4)	−1.3 (0.61)
Hassan 2012	Metformin	100.89	NA	NA	231.56	NA	NA	5.4 (0.47)	5.7 (0.47)	0.3 (0.47)
	Insulin	102.11	NA	NA	236.41	NA	NA	5.19 (0.59)	5.37 (0.48)	0.18 (0.543)
Spaulonei 2013	Metformin	102.15 (21.96)	90.09 (16.29)	−12.06 (19.75)	124.17 (24.4)	108.44 (13.39)	−15.73 (21.16)	5.9 (0.75)	NA	NA
	Insulin	100.87 (15.05)	88.35 (7.45)	−12.52 (13.03)	125.39 (22.12)	112.32 (13.69)	−13.07 (19.34)	5.93 (0.8)	NA	NA
Tertti 2013	Metformin	99 (9)	NA	NA	149.4 (32.4)	NA	NA	5.48 (0.34)	5.68 (0.33)	0.2 (0.34)
	Insulin	100.8 (7.2)	NA	NA	142.2 (30.6)	NA	NA	5.51 (0.34)	5.69 (0.36)	0.18 (0.35)
Ashoush 2016	Metformin	105.7 (4.7)	78 (3.1)	−27.7 (4.14)	175.7 (10)	109.9 (3.7)	−65.8 (8.757)	5.7 (0.5)	NA	NA
	Insulin	106.4 (4.4)	79.9 (3.7)	−26.5 (4.1)	177.6 (8.8)	111.3 (4.2)	−66.3 (7.624)	5.8 (0.6)	NA	NA
Saleh 2016	Metformin	136.09 (39.85)	93.23 (13.7)	−42.86 (35.07)	198.32 (214.67)	116.52 (3.53)	−81.8 (212.927)	NA	NA	NA
	Insulin	137.56 (41.1)	94.33 (11.11)	−43.23 (36.82)	196.52 (15.45)	117.12 (3.45)	−79.4 (14.05)	NA	NA	NA
Arshad 2017	Metformin	104.4 (13.12)*	93.48 (11.9)	−10.92 (12.55)	NA	NA	NA	5.28 (0.42)	5.42 (0.346)	0.14 (0.388)
	Insulin	117.9 (29.06)*	102.08 (20.63)	−15.82 (25.9)	NA	NA	NA	5.43 (0.34)	5.72 (0.35)	0.29 (0.345)
Gamal 2018	Metformin	NA	NA	NA	NA	NA	NA	6.9 (0.4)	6.5 (0.4)	−0.4 (0.4)
	Insulin	NA	NA	NA	NA	NA	NA	6.7 (0.5)	6.4 (0.4)	−0.3 (0.46)
Wasim 2019	Metformin	117 (18.1)	92.1 (6)	−24.9 (15.97)	NA	NA	NA	6.99 (7.5)	6 (0.9)	− 0.99 (7.09)
	Insulin	120 (22.4)	96.6 (6.2)	−23.4 (20.03)	NA	NA	NA	7.1 (0.79)	6.1 (1.1)	−1 (0.98)
Langer 2000	Glyburide	104 (25)	98 (13)	−6 (21.7)	174 (31)	113 (22)	−61 (27.6)	5.7 (1.3)	5.5 (0.7)	−0.2 (1.13)
	Insulin	108 (26)	98 (16)	−10 (22.7)	174 (39)	112 (15)	−62 (34.1)	5.6 (1.2)	5.4 (0.6)	−0.2 (1.04)
Anjalakshi 2007	Glyburide	NA	NA	NA	167.1 (22.97)	95.29 (7.41)	−71.81 (20.31)	5.48 (0.79)	5.3 (0.34)	−0.18 (0.686)
	Insulin	NA	NA	NA	174.92 (31.05)	93 (9.75)	−81.92 (27.5)	5.75 (1.23)	5.5 (0.62)	−0.25 (1.065)
Lain 2009	Glyburide	100.9 (15.9)	90.4 (21.8)	−10.5 (19.53)	176.9 (35.9)	109.8 (21.8)	−67.1 (31.33)	5 (0.5)	NA	NA
	Insulin	101.5 (12.4)	90.9 (7)	−10.6 (10.77)	173.1 (34.9)	106 (14)	−67.1 (30.42)	5 (0.5)	NA	NA
Mukhopadhyay 2012	Glyburide	103.5 (14.62)	88.23 (6.55)	−15.27 (12.68)	184.1 (20.46)*	122.7 (10.3)	−61.4 (17.72)	6.25 (0.6)	6.46 (0.77)	−0.17 (0.577)
	Insulin	109.3 (19.63)	88.17 (8.44)	−21.13 (17.06)	194.3 (18.47)*	128 (12.38)	−66.3 (16.3)	6.46 (0.77)	6.24 (0.57)	−0.22 (0.692)
Mirzamoradi 2015	Glyburide	109.83 (68.99)	114.02 (10.65)	4.19 (64.33)	NA	115.46 (8.21)	NA	NA	NA	NA
	Insulin	112.15 (19.39)	123.42 (14.71)	11.27 (17.53)	NA	120.15 (9.56)	NA	NA	NA	NA
Silva 2012	Glyburide	94.04 (16.25)	88.23 (11.71)	−5.81 (14.52)	160.83 (18.6)	126.44 (16.91)	−34.39 (17.81)	NA	5.62 (0.85)	NA
	Metformin	95.84 (20.91)	90.52 (11.78)	−5.32 (18.16)	165.59 (21.9)	126.48 (20.51)	−39.11 (21.18)	NA	5.51 (0.78)	NA
George 2015	Glyburide	100.8 (14.4)	86.4 (24.4)	−14.4 (21.24)	181.8 (45)	119.4 (21.25)	−62.4 (38.99)	5.9 (0.5)	NA	NA
	Metformin	102.6 (14.4)	88.2 (10.8)	−14.4 (12.98)	194.4 (46.8)	123.6 (15.41)	−70.8 (41.31)	5.8 (0.6)	NA	NA
Nachum 2017	Glyburide	95.9 (10.4)	88.7 (10.2)	−7.2 (10.3)	127.6 (19.1)	115.3 (13.8)	−12.3 (17.08)	NA	NA	NA
	Metformin	96.8 (10.5)	91.3 (8.8)	−5.5 (9.76)	125.4 (12.8)	112.6 (12.3)	−12.8 (12.56)	NA	NA	NA

FBG fasting blood glucose, 2HPG 2-h postprandial blood glucose, HbA1c hemoglobin A1c, SD standard deviation

* *P* value < 0.05, marked in bold

### FBG

The results of the pairwise meta-analysis of glycemic control are shown in Supplementary Figs. 4, 5 ,6, 7, 8, 9. The reduction in FBG from baseline associated with glyburide treatment was significantly smaller than that associated with insulin treatment (SMD 0.18; 95% CI 0.01, 0.34) (supplementary Fig. 4). However, after removal the study with imbalanced baseline level of 2HPG [57], the reduction in FBG from baseline showed no significant difference between glyburide and insulin treatment (SMD 0.15; 95% CI -0.03, 0.33)(supplementary Fig. 5). Moreover, the reduction in FBG from baseline did not significantly differ between the metformin and insulin groups (SMD -0.05; 95% CI − 0.15, 0.05) or the glyburide and metformin groups (SMD − 0.05; 95% CI − 0.23, 0.13) (supplementary Fig. 4). The NMA revealed no significances between metformin and insulin, glyburide and insulin, or glyburide and metformin (Table 3 and supplementary Table 2). Table 4 shows the treatment rankings, in which “1” is the least effective and “3” is the most effective. Metformin had the highest probability of being the least effective treatment for the reduction of FBG (62%), while insulin had the highest probability of being the most effective treatment (73%).

**Table 3:** Network meta-analysis of glycemic control and birth weight

FBG			2HPG			HbA1c			Birth weight		
Glyburide			Glyburide			Glyburide			Glyburide		
4.98	Insulin		2.56	Insulin		0.03	Insulin		0.05	Insulin	
(−9.46, 19.34)			(−3.24, 8.77)			(−0.17, 0.22)			(−0.02, 0.14)		
−2.49	−7.52	Metformin	4.45	1.81	Metformin	0.04	0.02	Metformin	**0.12**	**0.07**	Metformin
(−16.60, 11.81)	(− 18.61, 3.62)		(− 1.68, 10.42)	(−4.30, 7.51)		(−0.17, 0.25)	(−0.06, 0.10)		**(0.05, 0.21)**	**(0.01, 0.13)**	

Data are reported as mean difference (95% confidence interval) and indicate column-to-row difference. Statistically significant differences are in bold

**Table 4:** Ranking probability of the efficiency of different treatments

		Glyburide			Metformin			Insulin		
		**Worst Best**			**Worst Best**			**Worst Best**		
		**Rank1**	**Rank2**	**Rank3**	**Rank1**	**Rank2**	**Rank3**	**Rank1**	**Rank2**	**Rank3**
**Glycemic control**	**FBG**	0.34	**0.44**	0.22	**0.62**	0.33	0.05	0.04	0.23	**0.73**
	**2HPG**	**0.79**	0.18	0.04	0.05	0.23	**0.72**	0.17	**0.59**	0.24
	**HbA1c**	**0.57**	0.13	0.30	0.16	0.36	**0.48**	0.27	**0.51**	0.21
**Neonatal outcomes**	**Anomaly**	**0.78**	0.10	0.12	0.11	0.31	**0.58**	0.11	**0.59**	0.30
	**Hyperbiliru**	**0.86**	0.10	0.04	0.03	0.29	**0.68**	0.11	**0.61**	0.28
	**LGA**	**0.94**	0.05	0.01	0.01	0.19	**0.80**	0.05	**0.76**	0.19
	**Macrosomia**	**0.96**	0.04	0.00	0.00	0.05	**0.95**	0.04	**0.91**	0.05
	**NICU admission**	0.24	0.31	0.45	0.08	0.41	**0.51**	**0.67**	0.28	0.04
	**Neonatal hypoglycemia**	**0.94**	0.05	0.00	0.00	0.04	**0.95**	0.05	**0.90**	0.04
	**Obstetric trauma**	0.38	0.24	0.38	0.25	0.32	**0.44**	0.37	**0.44**	0.19
	**Perinatal death**	**0.59**	0.27	0.14	0.11	0.24	**0.66**	0.31	**0.49**	0.20
	**Preterm birth**	0.35	0.35	0.30	**0.58**	0.35	0.07	0.07	0.30	**0.63**
	**RDS**	0.26	0.23	**0.50**	0.28	**0.37**	0.35	**0.46**	0.39	0.15
	**SGA**	0.42	0.11	**0.47**	**0.48**	0.39	0.12	0.10	**0.50**	0.41
	**Birth weight**	**0.91**	0.09	0.00	0.00	0.01	**0.99**	0.09	**0.90**	0.01
**Preeclampsia**		**0.60**	0.29	0.11	0.01	0.12	**0.87**	0.39	**0.59**	0.02

LGA large-for-gestational age, SGA small-for-gestational age, RDS respiratory distress syndrome, NICU neonatal intensive care unit

The probability (based on Bayesian analysis) of treatment being the worst (rank 1) or the best (rank 3). Bold indicates the probability of different treatment that are most likely to be ranked

### 2HPG

Pairwise meta-analysis of the change in 2HPG from baseline showed heterogeneity in the trials that compared metformin with insulin (I^2^, 68%). The 2HPG reduction in the metformin group was larger than that in the glyburide group (*p* = 0.05; SMD 0.18; 95% CI 0, 0.36) (Supplementary Fig. 6). The 2HPG reduction in the glyburide and insulin groups were comparable before and after removal of study with imbalanced baseline (Supplementary Fig. 6 and 7). The NWA revealed no significant differences between the changes in 2HPG associated with each treatment (Table 3). With regard to the ranking probability, glyburide had the highest probability of being the least effective treatment (72%), while metformin had the highest probability of being the most effective treatment (79%) (Table 4).

### HbA1c

Pairwise meta-analysis of the change in HbA1c from baseline showed no significance differences between metformin and insulin, or glyburide and insulin (Supplementary Fig. 8 and 9). The NWA revealed no significant differences between the change in HbA1c from baseline associated with each treatment (Table 3). Glyburide had the highest probability of being the least effective treatment (57%) (Table 4).

### Neonatal outcomes

The most frequent adverse outcome reported was LGA with incidence ranging from 9.28 to 26.25%. The second most frequent neonatal outcome is neonatal hypoglycemia, with incidence ranging from 0.67 to 20.00%. Conversely, the most rarely adverse outcomes are perinatal death (0.00–4.00%), RDS (0.00–9.25%), obstetric trauma (0.00–4.50%) and anomaly (0.00–5.00%) (supplementary Table 3).

#### Neonatal hypoglycemia

**Fig. 1:**
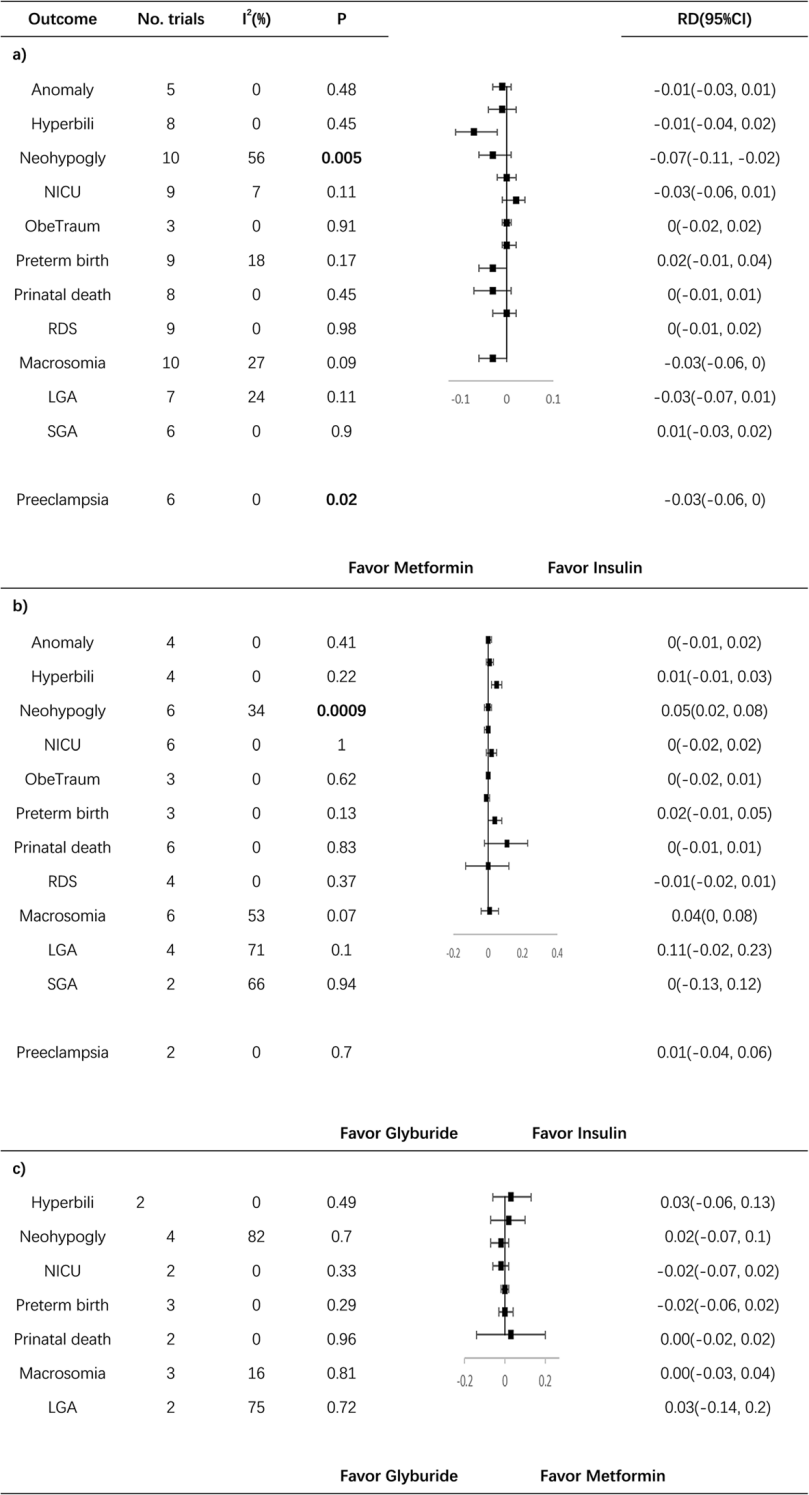
Pairwise meta-analysis of different treatments on neonatal outcomes and preeclampsia. Hyperbili hyperbilirubinemia, Neohypogly neonatal hypoglycemia, ObeTraum obstetric trauma. Statistically significant differences (*p* value < 0.05) are in bold

Pairwise meta-analysis showed a significant lower prevalence of neonatal hypoglycemia in the metformin group than in the insulin group (RD − 0.07; 95% CI − 0.11, − 0.02) (Fig. 1). Moreover, there was higher prevalence in the glyburide group than in the insulin group (RD 0.05, 95% CI 0.02, 0.08). However, no significant difference was found between the glyburide and metformin groups, which may be because of the heterogeneity in the trials (I^2^, 82%). Metformin had the highest probability of being the best option (95%), while the glyburide had the highest probability of being the worst option (94%), and insulin had the highest probability of ranking second (90%) (Table 4).

#### NICU admission

Pairwise meta-analysis showed no significant differences between metformin and inslin, glyburide and insulin or glyburide and metformin (Fig. 1). While, there was lower prevalence of NICU admission in the metformin group than in the insulin group (RD − 0.03; 95% CI − 0.06, 0.01). Metformin had the highest probability of being the best option (51%), while insulin had the highest probability of being the worst option (67%) (Table 4).

#### Macrosomia

Pairwise meta-analysis of macrosomia showed a lower prevalence in the metformin group than in the insulin group (RD − 0.03; 95% CI − 0.06, 0), but the difference was not significant (*p* = 0.09) (Fig. 1). Furthermore, the prevalence of macrosomia was higher in the glyburide group than in the insulin group (RD 0.04; 95% CI 0, 0.08), but the difference was not significant (*p* = 0.07). No significant difference was found between the glyburide and metformin groups. In the NMA, metformin was the best option, with a probability of 95%, while glyburide had the highest probability of being the worst option (96%). Insulin had the highest probability of ranking second (91%) (Table 4).

#### Birth weight

Supplementary Fig. 10 shows the results of the pairwise meta-analysis of birth weight. The metformin group showed significantly lower birth weight than the insulin group (SMD − 0.17; 95% CI − 0.25, − 0.08) and the glyburide group showed significantly higher birth weight than the insulin group (SMD 0.10; 95% CI 0.01, 0.20). The pairwise meta-analysis of glyburide and metformin showed high heterogeneity (I^2^, 72%) and no significant difference was detected. The NWA results were consistent with those of the pairwise meta-analysis (Table 3). Metformin had the highest probability of being the best option for the control of birth weight (99%), while glyburide had the highest probability of being the worst option (91%). Insulin had the highest probability of ranking second (90%) (Table 4).

#### Other neonatal outcomes

No significant differences were identified in the pairwise meta-analyses of anomalies, neonatal hyperbilirubinemia, obstetric trauma, perinatal death, preterm birth, RDS, LGA, or SGA (Fig. 1).

### Maternal outcomes

#### Preeclampsia

Pairwise meta-analysis of PE showed significantly lower prevalence in the metformin group than in the insulin group (RD − 0.03; 95% CI − 0.06, 0) (Fig. 1). However, no significant differences were found between glyburide and insulin or glyburide and metformin. The NMA revealed that metformin had the highest probability of being the most effective treatment for the prevention of PE (87%), while glyburide had the highest probability of being the least effective treatment (60%). Insulin had the highest probability of ranking second (59%) (Table 4).

#### Maternal weight gain

The studies of maternal weight gain had only compared metformin with insulin. Therefore, no NMA was conducted. The pairwise meta-analysis showed lower maternal weight gain after enrollment in the metformin group than in the insulin group (SMD − 0.61; 95% CI − 0.86, − 0.35) (Supplementary Fig. 11).

## Discussions

The present network meta-analysis included 23 comparisons of 4533 individuals with GDM who had been randomly assigned to glyburide, metformin, or insulin treatment. The results demonstrate that metformin is as effective as insulin for glycemic control, and is the most promising drug for the prevention of neonatal and maternal complications among the treatments compared. However, glyburide seems to be inferior to both insulin and metformin with respect to glycemic control, which may also explain the poorer neonatal outcomes that were associated with the use of this drug.

Metformin seems to be better than glyburide and no worse than insulin with respect to glycemic control. Moreover, metformin had the highest probability of being the most effective drug for the control of 2HPG among the three treatments evaluated, according to the NMA. This finding is consistent with the mechanisms of action of metformin, which increases insulin sensitivity and glucose uptake into muscle, liver, and adipose tissue [72]. Glyburide was significantly less effective at reducing FBG than insulin and 2HPG than metformin. However, no significant difference was found for the control of HbA1c.

The metformin group had lower prevalences of neonatal hypoglycemia, macrosomia, and NICU admission, while the glyburide group had a higher prevalence of neonatal hypoglycemia than insulin. This result was consistent with the effects of the drugs on glycemic control, and suggests that maternal blood glucose control, and especially 2HPG control, may be an important predictor of the neonatal outcomes of hypoglycemia, macrosomia, and NICU admission. However, no significant differences were found in the prevalences of severe neonatal conditions, such as anomalies, perinatal death, or RDS, between the use of OHAs and insulin. NMA showed that metformin had the highest probability of being the safest treatment for the prevention of the neonatal complications of macrosomia (95%), neonatal hypoglycemia (97%), LGA (80%), neonatal hyperbilirubinemia (76%), perinatal death (67%), NICU admission (60%), and anomalies (58%). This suggests that metformin is a safe treatment with respect to short-term neonatal outcomes.

Regarding the long-term outcomes, previous studies have shown that GDM is associated with obesity and diabetes in offspring [73–77]. Intrauterine hyperglycemia can result in epigenetic and structural alterations of fetal tissues, which are proposed mechanisms for the fetal origin of adult diseases [78–80]. Thus, better glycemic control is thought to be associated with superior long-term outcomes. However, because both of the OHAs are thought to pass across the placenta, the direct effects of metformin and glyburide on fetal development require investigation. As stated above, the metformin group had significant lower birth weight and a lower prevalence of macrosomia than the insulin group. Ijas et al. reported no significant difference in the ponderal index between the 18-month-old children of metformin- and insulin-treated mothers. Moreover, they also showed that the children of metformin-treated mothers were taller and heavier than those of insulin-treated mothers [42]. Rowan et al. reported larger amounts of subcutaneous fat in the 2-year-old children of metformin-treated mothers than in the children of insulin-treated mothers [44]. Furthermore, Wouldes et al. found that the children of metformin-treated mothers had similar neurodevelopmental outcomes to those of insulin-treated mothers at 2 years of age [43]. However, because of the limited number of studies conducted and the differing follow-up periods, it is not feasible to carry out meta-analyses of these outcomes. Moreover, studies with a longer follow-up period are required because some of the diseases that have fetal origins have onsets in early adulthood or at an even later stage.

Recent meta-analyses studies have compared the safety and efficiency of OHAs and insulin [37, 38]. No significant differences were identified in the glycemic control achieved using OHAs and insulin, and high heterogeneity were detected among the included trials. In the present meta-analysis, the heterogeneity among the studies was lower than in previous analyses. The present study design was different to that of previous meta-analyses in 3 principal respects. Firstly, the changes from baseline in FBG, 2HPG, HbA1c during treatment were selected as the primary outcomes, rather than the FBG, 2HPG, HbA1c levels after treatment. As GDM can be influenced by genetic backgrounds, which may differently affect blood glucose level among populations. Big differences of baseline FBG were reported among these studies, ranging from 96 to 172 mg/dl. In our study, after adjusting the data with baseline blood glucose, the effects of differences among populations were reduced. Secondly, studies that used a PP protocol and those with significantly different baseline maternal ages and gestational ages at enrollment were excluded. The studies that used a PP protocol excluded patients with inadequate glycemic control, which meant that patients with more serious disease were excluded. Ainuddin, J. et al. reported higher postprandial blood glucose in patients failed to achieve blood glucose control by metformin alone [45]. In this way, PP protocol would likely have resulted in inconsistencies when data from these studies were pooled with those from ITT studies. Thirdly, our study conducted both network and pairwise meta-analysis. The two most recently published meta-analyses were both pairwise meta-analysis. Guo, LL et al. reported higher prevalence of preeclampsia, NICU admission and neonatal hypoglycemia and macrosomia in insulin group compared with metformin group [38]. Meanwhile, the metformin group showed significantly lower weight gain during gestation compared with glyburide group [38]. Compared with insulin group, glyburide group had higher prevalence of neonatal hypoglycemia [38]. Our results of net-work meta-analysis combined the direct and indirect evidence and assessed the relative effectiveness of insulin, glyburide and metformin treatment in GDM. The results of our net-work meta-analysis suggested that metformin be the most promising intervention to reduce the incidence of neonatal hypoglycemia, LGA, macrosomia, hyperbilirubinemia, anomaly and perinatal death, while insulin and glyburide ranking second and third. Thus, our results are more likely to aid clinical decision-making.

Although there may have been statistical inconsistencies between the PP and ITT studies, the conclusions of the present meta-analysis were consistent with those of most of the PP studies. Mesdaghinia et al. reported a lower prevalence of macrosomia in metformin-treated mothers than insulin-treated mothers, and the prevalences of LGA, neonatal hyperbilirubinemia, RDS, and anomalies tended to be lower in the metformin group, although significant differences were not identified [55]. Ghomian et al. showed no significant differences in glycemic control, birth weight, or the prevalences of preterm birth or cesarean section between the metformin and insulin groups [70]. The prevalence of neonatal hypoglycemia tended to be lower in the metformin group, although this did not achieve significance [70]. Ruholamin et al. reported no significant differences in the prevalences of gestational complications, preterm birth, hyperbilirubinemia, NICU admission, macrosomia, SGA, or neonatal hypoglycemia between the metformin and insulin groups. However, the neonatal blood glucose concentrations 1 h and 2 h after birth were significantly lower in the insulin group than in the metformin group [61]. Finally, Behrashi et al. compared glyburide and insulin treatment and found that the glyburide group had a lower prevalence of macrosomia and lower birth weight than the insulin group. However, there were no differences in the prevalences of neonatal hypoglycemia, RDS, hyperbilirubinemia, anomalies, or NICU admission [65].

Two studies were excluded because of poor matching of the groups at baseline. Because their sample sizes were relatively small, significant differences at baseline can confound assessments of the severity of the disease or lead to different treatment durations in the two groups, such that the comparisons would not be effectively controlled. The study reported by Ogunymi et al. was characterized by significantly different gestational ages at enrollment (metformin vs. insulin, 28.1 ± 7.6 weeks vs. 24.6 ± 8 weeks), and therefore this study was not included in the meta-analysis to avoid introducing bias, despite the fact that they reported similar results to those of the present study with respect to the effects of insulin and glyburide on 2HPG control and the prevalence of neonatal hypoglycemia [51]. Similarly, the study reported by Mirzamoradi et al. was not included because of significantly different maternal age in the glyburide and insulin groups [63].

The prevalence of treatment failure was also compared between the glyburide and metformin groups, but no significant difference was found (Supplementary Fig. 12). However, because the two drugs regulate blood glucose through different mechanisms, glyburide may be effective in patients who do not respond to metformin [16, 17]. Thus, glyburide may represent an alternative treatment for patients who are not willing to administer daily injections and frequently monitor their blood glucose.

Almost all of the included studies were conducted in urban areas. However, patients in rural areas may be more likely to have a lower educational level and be less affluent, which can result in non-compliance and inadequate glucose monitoring during insulin treatment [81, 82]. Therefore, the effectiveness of insulin treatment may be overestimated for patients in rural areas. To provide complete evidence for decision makers, the cost-benefit ratio is a very important index of the utility of a therapy. However, the mean dosage of drug administered and their costs were missing from most of the studies. Moreover, because of the different methods of administration of OHAs and insulin, it is impossible to blind the patients, and therefore all the studies comparing the effects of OHA and insulin had a risk of bias. Last but not least, most of the studies were conducted with modest sample sizes; therefore, differences between groups may have been missed due to a lack of power.

In conclusion, the present study provides evidence that metformin is as effective as insulin for glycemic control in GDM and is superior with respect to the prevention of adverse neonatal outcomes, such as neonatal hypoglycemia, macrosomia, and NICU admission. However, further studies with larger sample sizes are required to assess the long-term effects of metformin treatment on offspring outcomes and to determine the cost-benefit ratios of the use of the drugs in rural populations.

## Supplementary Information

**Additional file 1: Supplementary Table 1.** Study and patient characteristics of included trials. TTN: transient tachypnea of newborn.

**Additional file 2: Supplementary Fig. 1.** Flow chart of study selection.

**Additional file 3: Supplementary Fig. 2.** Risk of bias graph.

**Additional file 4: Supplementary Fig. 3.** Risk of bias summary.

**Additional file 5: Supplementary Fig. 4.** Pairwise meta-analysis of FBG.

**Additional file 6: Supplementary Table 5.** Pairwise meta-analysis of FBG after removal studies with imbalanced baseline blood glucose.

**Additional file 7: Supplementary Fig. 6.** Pairwise meta-analysis of 2HPG.

**Additional file 8: Supplementary Table 7.** Pairwise meta-analysis of 2HBG after removal studies with imbalanced baseline blood glucose.

**Additional file 9: Supplementary Fig. 8.** Pairwise meta-analysis of HbA1c.

**Additional file 10: Supplementary Table 9.** Pairwise meta-analysis of HbA1c after removal studies with imbalanced baseline blood glucose.

**Additional file 11: Supplementary Table 2.** Network meta-analysis of glycemic control and birth weight.

**Additional file 12: Supplementary Fig. 10.** Pairwise meta-analysis of birth weight.

**Additional file 13: Supplementary Fig. 11.** Pairwise meta-analysis of maternal weight gain.

**Additional file 14: Supplementary Fig. 12.** Meta-analysis of treatment failure rate.

**Additional file 15: Supplementary Table 3.** Prevalence of adverse outcome.

## Data Availability

All data generated or analysed during this study are included in this published article and its supplementary information files.
