# Supplementary material for: Glycemic control and neonatal outcomes in women with gestational diabetes mellitus treated using glyburide, metformin, or insulin: a pairwise and network meta-analysis
Source: BMC Endocr Disord. 2021 Oct 12;21:199. doi: 10.1186/s12902-021-00865-9 (PMC8513183; doi:10.1186/s12902-021-00865-9)
Supplement: Supplementary file 1 — Additional file 1: Supplementary Table 1. Study and patient characteristics of included trials. TTN: transient tachypnea of newborn. [file 12902_2021_865_MOESM1_ESM.docx]

| **Study (author year)** | **Screen** | **Diagnose** | **Primary outcome** | **Funding** | **Conflict of interest** | **Glycemic control target** | **Insulin** |
| --- | --- | --- | --- | --- | --- | --- | --- |
| Rowan  2000 | ADIPS: 1h 50g GCT≥7.8mmol/l, or 1h 75g GCT≥8.0mmol/l | ADIPS: 75g 2h OGTT: FBG≥5.5mmol/l or 2h ≥8.0mmol/l | NA | NA | No | NA | NA |
| Moore  2007 | 1h 50g GCT≥140mg/dl | ADA: 3h OGTT FBG:105mg/dl, 1h: 190mg/dl, 2h: 165mg/dl, 3h: 145mg/dl, 2 or more elevated values. | FBG, 2HPG, mode of delivery, incidence of shoulder dystocia, postpartum hemorrhage | NA | No | FBG<105 mg/dl, 2HPG≤120 mg/dl | regular insulin and NPH |
| Rowan  2008 | ADIPS: 1h 50g GCT≥7.8mmol/l, or 1h 75g GCT≥8.0mmol/l | ADIPS: 75g 2h OGTT: FBG≥5.5mmol/l or 2h ≥8.0mmol/l | a composite of neonatal hypoglycemia, respiratory distress, need for phototherapy, birth trauma, 5-minute Apgar score less than 7, or prematurity | NA | No | FBG<97.2mg/dl, 2HPG≤120.7mg/dl | typically a short-acting insulin analog before meals and intermediate insulin once or twice daily |
| Ijas  2010 | Risk factors | 2h 75g: FBG 95.4, 1h 198. 2h 172.8mg/dl, ≥1 elevated values | incidence of macrosomia (>4000g) | The Foundation of Alma and K.A. Snellman, Oulu, Finland. | No | FBG<5.3mM, 1.5HPG≤6.7mM | long-acting insulin and rapid-acting insulin |
| Niromanesh  2012 | 1h 50g GCT≥130mg/dl | Carpenter Coustan: 3h 100g OGTT: FBG:95, 1h:18, 2h:155, 3h:140mg/dl, ≥2 elevated values | maternal glycemic control and birth weight | NA | No | FBG<95mg/dl, 2HPG≤120mg/dl | NPH + regular |
| Mesdaghinia  2012 | 1h 50g GCT | Carpenter Coustan: 3h 100g OGTT: FBG 95mg/dl, 1h 180mg/dl, 2h 155mg/dl, 3h 140mg/dl, ≥2 elevated values | NA | NA | No | FBG<95mg/dl, 2HPG≤120mg/dl | NPH + regular |
| Hassan  2012 | Risk factors, and 1h 50g GCT≥140mg/dl | WHO: 2h 75g OGTT: FBG>95, 1h≥180, 2h ≥155mg/dl, ≥2 elevated values | macrosomia | NA | No | FBG<100mg/dl, 1.5HPG≤126mg/dl | a combination of regular and intermediate acting Human insulin before meals twice daily |
| Spaulonei  2013 | NA | ADA: FBG 95, 1h 180, 2h 155, 3h 140mg/dl, ≥2 elevated values | glycemic control | NA | No | FBG≤95mg/dl, 2HPG≤120gm/dl | NPH + regular |
| Tertti  2013 | Risk factors | Finland national criteria: FBG≥4.8/5.3,1h≥10.0/10.0,2h≥8.7/8.6mmol/l (before/after 2008.12), 2 or more abnormal values | birth weight | NA | No | FBG<5.5mM, 1HPG<7.8mM | NPH insulin and rapid-acting insulin lispro or insulin aspart |
| Ruholamin  2014 | NA | ADIPS | NA | funded by Isfahan University of Medical Sciences | No | FBG<95mg/dl, 2HPG<120mg/dl | NA |
| Ashoush  2016 | NA | ADA: 2h 75g OGTT | successful glycemic control | NA | No | FBG<100mg/dl, 2HPG<140mg/dl | a mixture of regular insulin and NPH insulin before breakfast and before dinner |
| Saleh  2016 | 50g 1h GCT | WHO: 2h 75g OGTT: FBG≥7.0mmol/l or 2h >7.8mmol/l | NA | NA | No | FBG<100mg/dl, 2HPG<120mg/dl | a combination of short acting (Actrapid) and intermediate acting (Mixtard) human insulin as twice daily |
| Arshad  2017 | Risk factors | WHO criteria | NA | Self and Dow University of Health Sciences | NA | NA | 2/3 of the dose was prescribed in the morning and 1/3 of the dose was advised in the night |
| Gamal  2018 | 1h 50g GCT≥140mg/dl | 3h 100g OGTT: FBG>95mg/dl, 1h≥180, 2h ≥155, 3h≥140mg/dl, 2 or more abnormal values | control of diabetes mellitus monitored by fasting blood sugar level, two hour postprandial, and HbA1C | NA | NA | FBG≤100mg/dl, 2HPG≤126mg/dl | combination of short acting and intermediate acting twice daily |
| Ghomian  2019 | NA | NA | NA | Mashhad University of Medical Sciences; Research Council at the Mashhad University of Medical Sciences, Mashhad, Iran | No | FBG<95mg/dl, 2HPG<120mg/dl | three doses of short acting (insulin aspart) before each meal and long-acting (Levemir) before night sleep |
| Wasim  2019 | NA | IADPSG: 2h 75g OGTT FBG>92mg/dl and (1h≥180 or 2h ≥153mg/dl) | maternal glucose control, FBG, RBG at delivery | No funding required | No | FBG≤95mg/dl, 1HPG≤140mg/dl, HbA1c≤6% | three doses of short acting (Humulin R) before each meal & single dose of intermediate acting (NPH) at bed time |
| Langer  2000 | 1h 50g GCT>130mg/dl | 100g OGTT: 2 or more abnormal values | achievement of the desired level of glycemic control | NA | NA | Mean BG 5.0-5.9mM, FBG 3.4-5.0mM, preprandial BG 4.5-5.3mM, postprandial ≤6.7mM | rapid-action human insulin (Regular) three times daily |
| Bertini  2005 | FBG | WHO: 2h 75g OGTT FBG≥110mg/dl and 2h≥140mg/dl | NA | NA | NA | FBG≤90mg/dl, 2HPG≤100mg/dl | Regular before meals and NPH at bed times |
| Anjalakshi  2007 | NA | WHO: 2h 75g OGTT>140mg/dl | NA | NA | NA | NA | NA |
| Ogunyemi  2007 | NA | NA | maternal glycemic control and neonatal birth weight and outcomes | NA | No | NA | NPH + regular |
| Lain  2009 | 1h 50g GCT>135mg/dl | 1 of the 3 following: 1)FBG-95, 1h-180, 2h-155, 3h-140mg/dl, 2 or more abnormal values, 2)elevated 3h blood glucose, 3)1h >200mg/dl | percent of neonatal fat mass | NA | NA | FBG<95mg/dl, 2HPG<120mg/dl | long-acting and short acting insulin |
| Mukhopadhyay  2012 | NA | WHO: 2h 75g OGTT: 2h≥140mg/dl | NA | NA | No | Mean BG<105mg/dl, FBG<90mg/dl, 2HPG<120mg/dl | hree times daily |
| Tempe  2013 | 1h 50g GCT≥130mg/dl | 3h 100g OGTT: 2 or more abnormal values, or GCT≥200mg/dl | achievement of glycemic control | NA | No | FBG ≤95mg/dl, 2HPG≤120mg/dl, HbA1c≤6.5% | The lente and plain insulin were administered in the morning and at night |
| Mirzamoradi  2015 | NA | 2h OGTT: FBG>95mg/dl, 1h>180mg/dl, 2h>150mg/dl | effective glycemic index control | NA | NA | Mean BG 90-105mg/dl, FBG 60-90mg/dl, preprandial BG 90-95mg/dl, 2HPG<120mg/dl | NPH + regular |
| Bhrashi  2016 | FBG>92mg/dl | 3h 100g OGTT | NA(neonatal outcomes) | NA | No | FBG<90mg/dl, 2HPG<120mg/dl | twice per day 2/3 of the dose was prescribed in the morning and 1/3 in the evening; a combination of NPH and regular |
| Senat  2018 | NA | 2h 75g OGTT: FBG: 92mg/dl, 1h: 180mg/dl, 2h: 153mg/dl | a composite criterion including macrosomia, neonatal hypoglycemia, and hyperbilirubinemia | French Ministry of Health (PHRC 2011) and Département de la Recherche Clinique et du Développement de l’Assistance Publique–Hôpitaux de Paris | Dr Sentihes : consultancy work and lecturing for Ferring | FBG<95mg/dl, 2HPG<120mg/dl | basal + intermediate insulin |
| Moore  2009 | 1h 50g GCT≥130mg/dl | Carpenter Coustan: 3h 100g OGTT: FBG:95mg/dl, 1h:180mg/dl, 2h:155mg/dl, 3h:140mg/dl, 2 or more elevated values | glycemic control | NA | No | FBG≤105mg/dl, 2HPG≤120mg/dl | / |
| Silva  2012 | NA | WHO criteria | maternal glycemic control, birth weight and neonatal hypoglycemia | NA | No | FBG≤90mg/dl, 1HPG≤120mg/dl | / |
| George  2015 | Risk factors | 3h 100g OGTT: FBG≥5.3mmol/l, 1h≥10mmol/l, 2h≥8.6mmol/l, 3h≥7.8mmol/l, 2 or more elevated values | a composite of neonatal outcomes namely macrosomia, hypoglycaemia, need for phototherapy, respiratory distress, stillbirth or neonatal death and birth trauma | NA | NA | FBG≤5.3mM, 2HPG≤6.7mM | / |
| Nachum  2017 | NA | Carpenter Coustan or NDDG | the rate of treatment failure and glycemic control after the first-line medication according to mean daily glucose charts | NA | No | FBG≤95mg/dl, 1.5HPG≤130mg/dl | / |

Supplementary table 1. Study and patient characteristics of included trials. TTN: transient tachypnea of newborn.
