# Supplementary figures and images for: Glycemic control and neonatal outcomes in women with gestational diabetes mellitus treated using glyburide, metformin, or insulin: a pairwise and network meta-analysis
Source: BMC Endocr Disord. 2021 Oct 12;21:199. doi: 10.1186/s12902-021-00865-9 (PMC8513183; doi:10.1186/s12902-021-00865-9)

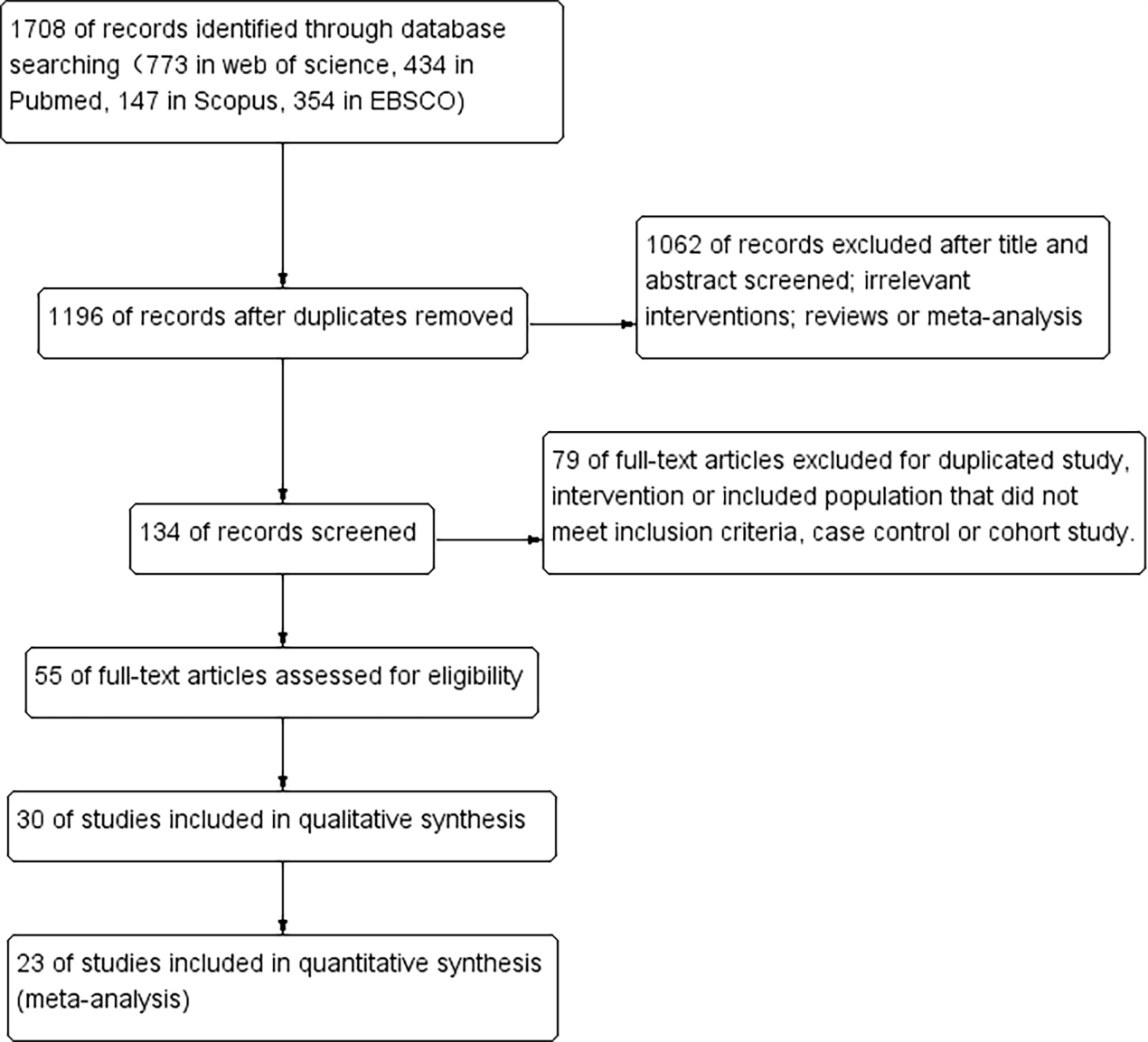


Supplementary figure 1. Flow chart of study selection.

Supplement: Supplementary file 2 — Additional file 2: Supplementary Fig. 1. Flow chart of study selection. [file 12902_2021_865_MOESM2_ESM.docx]

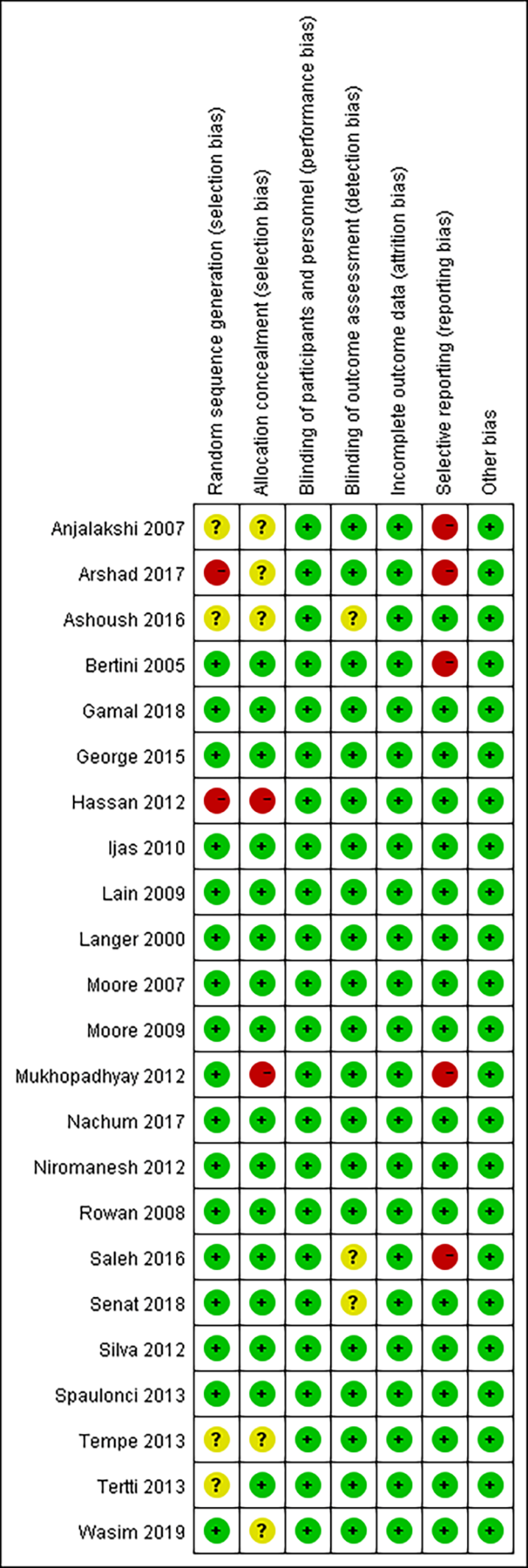


Supplementary figure 2. Risk of bias graph.

Supplement: Supplementary file 3 — Additional file 3: Supplementary Fig. 2. Risk of bias graph. [file 12902_2021_865_MOESM3_ESM.docx]

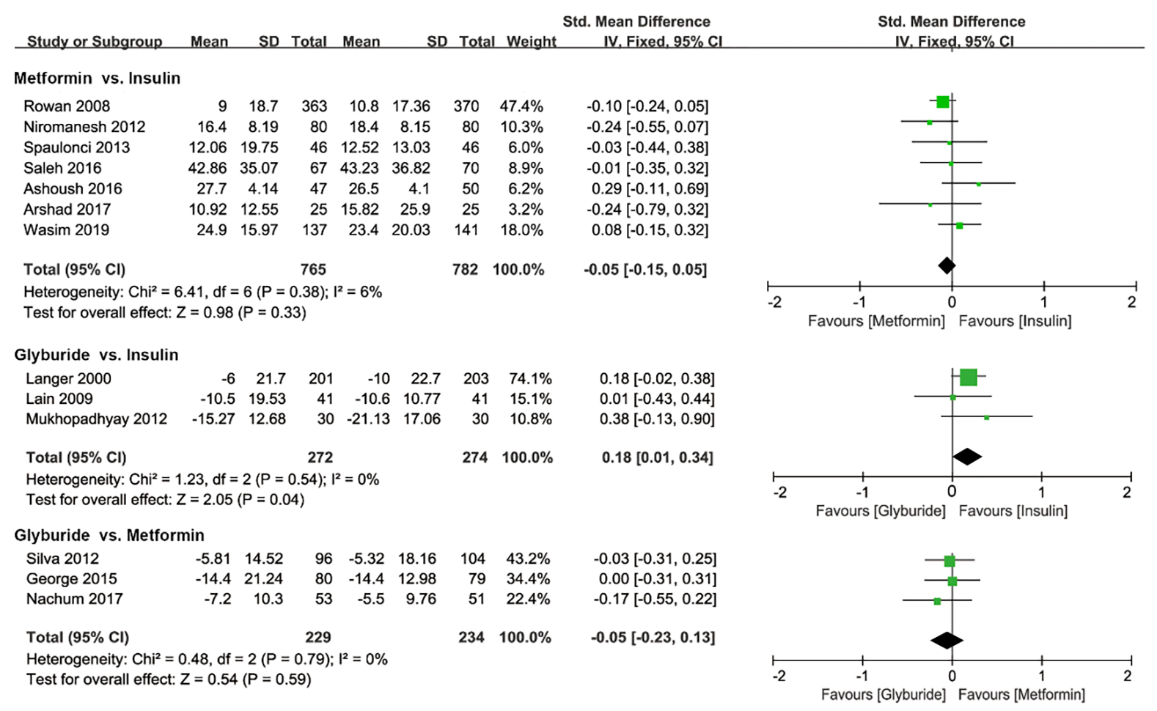


Supplementary figure 4. Pairwise meta-analysis of FBG.

Supplement: Supplementary file 5 — Additional file 5: Supplementary Fig. 4. Pairwise meta-analysis of FBG. [file 12902_2021_865_MOESM5_ESM.docx]

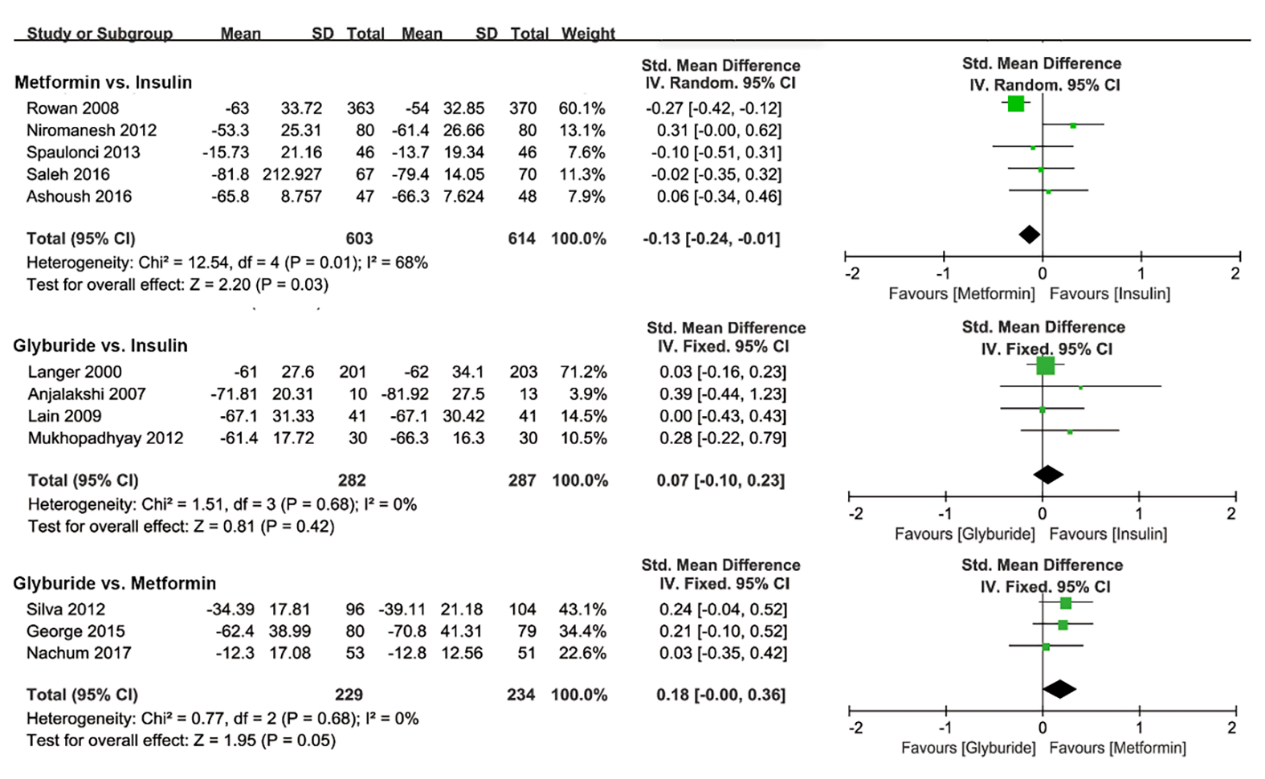


Supplementary figure 6. Pairwise meta-analysis of 2HPG.

Supplement: Supplementary file 7 — Additional file 7: Supplementary Fig. 6. Pairwise meta-analysis of 2HPG. [file 12902_2021_865_MOESM7_ESM.docx]

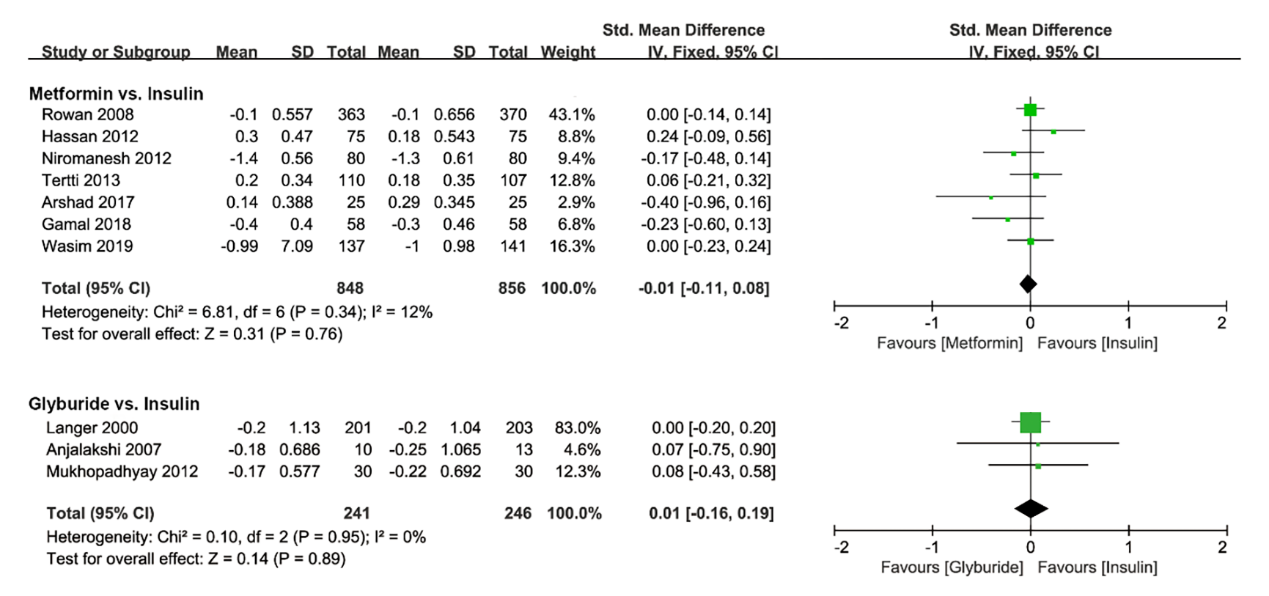


Supplementary figure 8. Pairwise meta-analysis of HbA1c.

Supplement: Supplementary file 9 — Additional file 9: Supplementary Fig. 8. Pairwise meta-analysis of HbA1c. [file 12902_2021_865_MOESM9_ESM.docx]

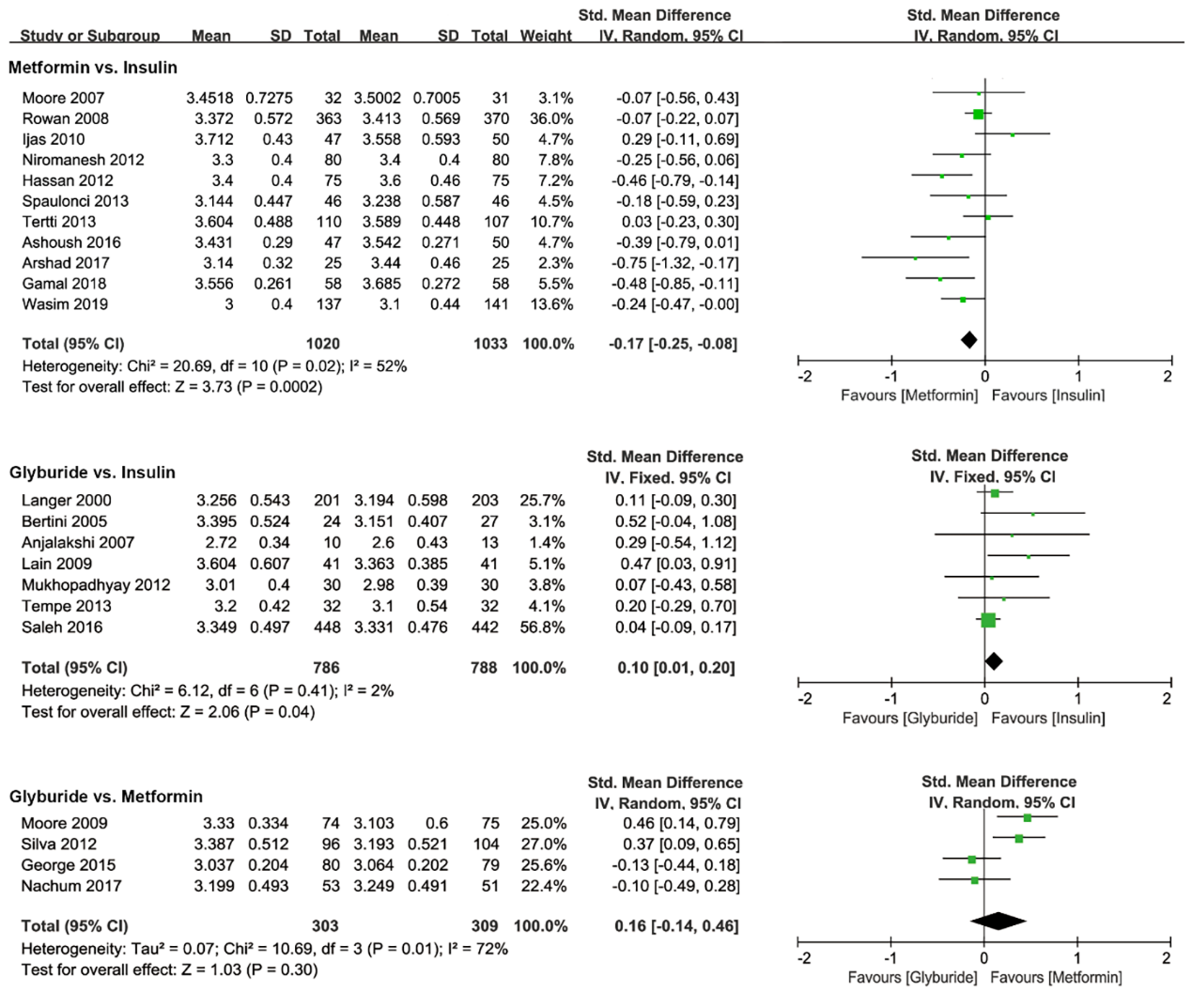


Supplementary figure 10. Pairwise meta-analysis of birth weight.

Supplement: Supplementary file 12 — Additional file 12: Supplementary Fig. 10. Pairwise meta-analysis of birth weight. [file 12902_2021_865_MOESM12_ESM.docx]

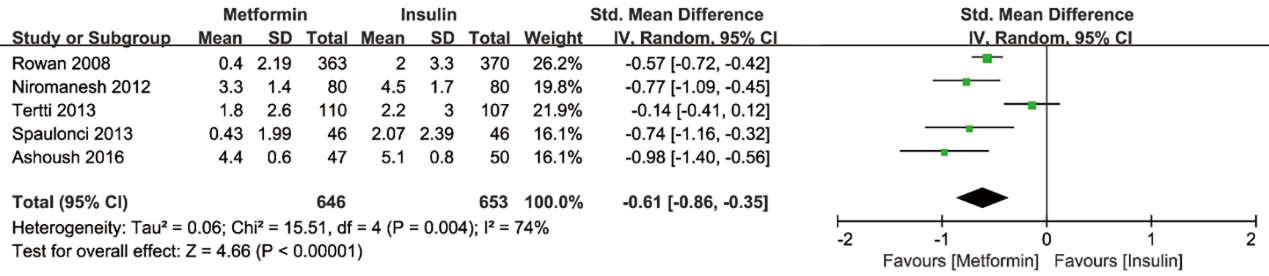


Supplementary figure 11. Pairwise meta-analysis of maternal weight gain.

Supplement: Supplementary file 13 — Additional file 13: Supplementary Fig. 11. Pairwise meta-analysis of maternal weight gain. [file 12902_2021_865_MOESM13_ESM.docx]
