# Supplementary material for: Glycemic control and neonatal outcomes in women with gestational diabetes mellitus treated using glyburide, metformin, or insulin: a pairwise and network meta-analysis
Source: BMC Endocr Disord. 2021 Oct 12;21:199. doi: 10.1186/s12902-021-00865-9 (PMC8513183; doi:10.1186/s12902-021-00865-9)
Supplement: Supplementary file 4 — Additional file 4: Supplementary Fig. 3. Risk of bias summary. [file 12902_2021_865_MOESM4_ESM.docx]

Supplementary figure 3. Risk of bias summary.
