# Supplementary material for: Glycemic control and neonatal outcomes in women with gestational diabetes mellitus treated using glyburide, metformin, or insulin: a pairwise and network meta-analysis
Source: BMC Endocr Disord. 2021 Oct 12;21:199. doi: 10.1186/s12902-021-00865-9 (PMC8513183; doi:10.1186/s12902-021-00865-9)
Supplement: Supplementary file 11 — Additional file 11: Supplementary Table 2. Network meta-analysis of glycemic control and birth weight. [file 12902_2021_865_MOESM11_ESM.docx]

| Supplementary table 1. Network meta-analysis of glycemic control and birth weight | | | | | | | | |
| --- | --- | --- | --- | --- | --- | --- | --- | --- |
| **FBG** | | | **2HPG** | | | **Birth weight** | | |
| Glyburide |  |  | Glyburide |  |  | Glyburide |  |  |
| 5.29 | Insulin |  | 1.75 | Insulin |  | 0.05 | Insulin |  |
| (-9.46,20.47) |  |  | (-4.84, 9.16) |  |  | (-0.02,0.14) |  |  |
| -2.47 | -7.80 | Metformin | 3.91 | 2.14 | Metformin | **0.12** | **0.07** | Metformin |
| (-17.33,12.38) | (-20.34,4.27) |  | (-2.58, 10.45) | (-4.61, 8.33) |  | **(0.05,0.21)** | **(0.01,0.13)** |  |

Data are reported as mean difference (95% confidence interval) and indicate column-to-row difference. Statistically significant differences are in bold
