# Supplementary material for: Glycemic control and neonatal outcomes in women with gestational diabetes mellitus treated using glyburide, metformin, or insulin: a pairwise and network meta-analysis
Source: BMC Endocr Disord. 2021 Oct 12;21:199. doi: 10.1186/s12902-021-00865-9 (PMC8513183; doi:10.1186/s12902-021-00865-9)
Supplement: Supplementary file 15 — Additional file 15: Supplementary Table 3. Prevalence of adverse outcome. [file 12902_2021_865_MOESM15_ESM.docx]

| Supplementary table 3. Prevalence of adverse outcome. | | | |
| --- | --- | --- | --- |
| Adverse event | Min(%) | Max(%) | Overall(%) |
| Preeclampsia | 3.36 | 18.25 | 11.32 |
| Perinatal death | 0.00 | 4.00 | 0.83 |
| Preterm birth | 0.00 | 11.87 | 8.22 |
| Neonatal hypoglycemia | 0.67 | 20.00 | 12.31 |
| NICU admission | 1.67 | 22.00 | 9.16 |
| RDS | 0.00 | 9.52 | 2.66 |
| Obstetric trauma | 0.00 | 4.50 | 2.23 |
| Hyperbiliru | 0.00 | 33.30 | 8.71 |
| Anomaly | 0.00 | 5.00 | 2.08 |
| Macrosomia | 0.00 | 22.10 | 11.19 |
| LGA | 9.28 | 26.25 | 16.47 |
| SGA | 2.44 | 8.45 | 7.24 |

Min: the lowest prevalence reported of the studies included. Max: the highest prevalence reported of the studies included. Overall: the average incidence of all studies included reporting this outcome. Overall prevalence=(the sum of events of all studies)/(the sum of individuals of all studies).
